# International best-practice models for perinatal and infant mental health care – a scoping review

**DOI:** 10.3389/fpsyt.2025.1536145

**Published:** 2025-06-17

**Authors:** Inanna Reinsperger, Jean Lillian Paul, Ingrid Zechmeister-Koss

**Affiliations:** ^1^ HTA Austria - Austrian Institute for Health Technology Assessment GmbH, Vienna, Austria; ^2^ Division of Psychiatry I, Department of Psychiatry, Psychotherapy, Psychosomatics, and Medical Psychology, Medical University Innsbruck, Innsbruck, Austria

**Keywords:** perinatal mental health, pregnancy, postpartum period, integrated care, care models, evidence-based guidelines

## Abstract

**Background:**

Perinatal mental illnesses (PMI) affect up to 20% of women and 10% of men during pregnancy and in the first year after the birth of the child. Perinatal mental illness contributes significantly to maternal mortality and adverse neonatal, infant, and child outcomes. Because of the high prevalence and the impact of PMI on both the parents and the infant, there is an urgent need for rapid and effective care. The aim of this scoping review was to identify comprehensive evidence-based guidelines and care models for the prevention and treatment of PMI and summarize their common characteristics.

**Methods:**

We searched manually in several databases and on websites of relevant institutions and contacted experts. We included guidelines and guidance documents based on pre-defined inclusion criteria.

**Results:**

We identified six relevant guidelines and care models from four countries (United Kingdom, Ireland, Canada, Australia). The identified documents highlight the need for integrated care models (including prevention, early identification, counseling, treatment), clear referral pathways, stepped-care approaches and multi-professional, coordinated networks.

**Conclusions:**

The ‘ideal’ care model should consider not only the mental health of the mother, but also that of the father/co-parent and the children, as well as the parent-infant relationship. The results from this scoping review can be used for further discussion and as decision support for designing, developing, and implementing perinatal and infant mental health (PIMH) care.

## Introduction

Mental illnesses are among the most common morbidities of the perinatal period, with potential serious adverse effects on the mother, the child, and the family (1). Overall, up to 20% of women are affected by perinatal mental illness (PMI) during pregnancy and the first year of life of the child (1, 2), with significant heterogeneity between countries (3). Depression is the most frequent PMI, with a prevalence of 12-18% according to systematic reviews (3, 4); followed by anxiety disorders, which affect 8-15% of women during the perinatal period (5, 6). Other mental illnesses, such as bipolar disorder [2.6% in women with no known previous psychiatric illness (7)], or postpartum psychosis [0.1-0.2% (8)] are less common. Partners may also experience perinatal mental health difficulties: between 5-10% of fathers are affected by perinatal depression and 5-15% experience perinatal anxiety; the prevalence of PMI in step-parents, co-mothers, trans and gender-diverse parents is still unknown (9). In up to 3.2% of couples, both parents experience perinatal depression at the same time (10).

PMI contributes significantly to maternal mortality and adverse neonatal, infant, and child outcomes (11). In very rare but extreme cases, PMI can lead to suicide and/or infanticide (12). Because of the high prevalence and the impact of PMI on both the parents and the infant, there is an urgent need for rapid and effective care (13, 14). There are several efforts on a global level to prioritize perinatal mental health. McNab et al. (15) proposed seven actions to promote perinatal mental wellbeing as a human right. This includes the integration of evidence-based approaches into health services and the capacity improvement of existing providers through training, supervision, and adequate remuneration. Another action proposed by McNab et al. is the strengthening of integrated care pathways between community and facilities and the reduction of stigma (15). Perinatal mental health services ideally include the prevention, detection, *and* management of PMI, including both new onset problems, recurrences of previous problems, as well as mental health problems already existing before conception (16). A recent systematic review of recommendations from member countries of the Organisation for Economic Co-operation and Development (OECD) has shown that most of the identified publications recommended routine screening for perinatal depression for all women. However, there was disagreement on various aspects, such as screening timing, responsible providers, setting, screening tool, and the follow-up and referral pathways that are required post-screening (17).

This scoping review further addresses this topic but takes a broad perspective by considering the whole care continuum from prevention to treatment and all perinatal mental illnesses. The review was conducted as a first step to identify international evidence for service models in order to subsequently co-develop and improve services in Tyrol (Austria) as part of the research project ‘Co-designing perinatal mental health support in Tyrol’ funded by the Austrian Science Fund. We aimed to provide an overview of international best-practice care models, pathways, and guidelines for perinatal and infant mental health (PIMH), and to analyze and describe their characteristics, as well as to highlight areas where open questions remain.

## Methods

A scoping review is a type of evidence synthesis that aims at identifying and mapping relevant evidence meeting pre-specified inclusion criteria. The review question of a scoping review is usually broader than that of a systematic review and scoping reviews may include different types of evidence. Their aim is typically to provide a comprehensive overview of the evidence rather than a quantitative or qualitative synthesis of data (18). The Preferred Reporting Items for Systematic reviews and Meta-Analyses extension for Scoping Reviews (PRISMA-ScR) Checklist was utilized as reporting guidance (19).

### Inclusion criteria

We included evidence-based guidelines and policy/guidance documents in English or German that describe care models for PIMH. We were interested in comprehensive care models that contained recommendations and information on various aspects of perinatal mental healthcare (prevention, screening, treatment, …) and excluded documents if they focused on one aspect only (e.g., screening), or if they only covered one specific indication (e.g., depression). We included documents that give recommendations on the ‘ideal’ content, organization and implementation of PIMH care models (not those describing the actual status quo in a country). As this review was conducted as part of a research project that aims to improve PIMH care in a part of Austria, we focused on countries from Europe. Additionally, we included documents from Australia and Canada, because we were aware that those countries were further progressed with PIMH care and could have helpful insights for the Austrian system. The inclusion criteria for relevant documents for this scoping review are summarized in **Table 1**.

**Table 1:** Inclusion and exclusion criteria for relevant documents.

Component	Inclusion criteria	Exclusion criteria
Population	Parents with a mental health problem during pregnancy and in the first year after birth and their infants	–
Intervention	Comprehensive perinatal and infant mental healthcare models and care pathways with information on various components of care (such as prevention, screening, treatment, …)	Models/pathways only focusing on one component of care (e.g., screening) Models/pathways dealing with care for mental illness outside the perinatal period
Categories of interest	Characteristics of care models: e.g., ▪ populations addressed ▪ professionals involved ▪ coordination ▪ ’components’ of the care models (e.g., primary prevention, screening, referral and assessment, treatment, specific services)	–
Study design	Policy/guidance documents, evidence-based guidelines	–
Setting	Europe, Canada, Australia	Other regions (e.g., Asia, Africa)
Languages	English, German	Other languages
Search period	Until July 2022	From July 2022 onwards

### Literature search and expert consultation

We conducted a comprehensive manual search for relevant PIMH care models as well as evidence-based guidelines. From May to July 2022, we searched in the following databases:

▪ PubMed,
▪ Google/Google Scholar,
▪ Guidelines International Network (G-I-N), and
▪ TRIP database (https://www.tripdatabase.com/).

For identifying relevant guidelines, we used the key words ‘perinatal mental healthcare’, ‘perinatal and infant mental healthcare’, ‘parental mental healthcare’ and, if applicable, restricted the search to ‘guidelines’. For the search of care models and pathways, we used the same key words and combined them with the terms ‘care model’, ‘service delivery model’, ‘(care) pathway’ and ‘integrated care’. The search strategy was tested and refined based on initial searches, to ensure specificity rather than an exhaustive systematic search, given the nature of the scoping review process. Additionally, we searched on websites from various relevant institutions. Details can be found in the **Supplementary Material**.

In addition, we contacted several relevant experts (e.g., from public health institutions or authors from articles on PIMH care), to ask for national models and relevant documents. Our request was also sent via the mailing list of the International Marcé Society for Perinatal Mental Health. The responses we received from members of the Marce Society provided valuable information on the topic, but did not result in a document that met our inclusion criteria.

We refrained from conducting a systematic literature search because we realized during the scoping process that the care models that we were interested in would rather be found in grey literature than in scientific articles.

### Literature selection

A pre-selection of potentially relevant documents was made by one researcher (IR) during the manual search. The final selection of documents was discussed by two researchers (IR and JP), based on the pre-defined inclusion criteria (see **Table 1**). Differences were resolved through the involvement of a third researcher (IZK).

### Data extraction and analysis

We prepared a data extraction table for each of the selected documents with predefined categories that were inductively expanded. We focused on the main characteristics of the PIMH care model described in the document/guideline (e.g., target populations, involved professionals, organization of the services, ‘components’ of the care models). The data extraction was performed by one researcher (IR) and checked for comprehensibility by a second researcher (JP). The full data extraction tables can be accessed via the authors on request. The information was summarized across all documents for each extracted category. The content of each category was qualitatively analyzed and narratively synthesized.

### Quality assessment

The quality of the included guidelines was assessed using the Appraisal of Guidelines for Research & Evaluation II (AGREE II) Instrument (20). As we did not identify a specific tool which can be used for the quality assessment of the documents describing the care models, we selected relevant criteria from the AGREE II instrument to get an impression of the quality of those documents. From the 23 items of the original tool, we chose 11 items that were also relevant for other types of documents. The item selection can be found in the **Supplementary Material**.

### Protocol

A protocol for this scoping review was developed *a priori* and published on the institution’s website (21).

## Results

### Included documents and general information

We identified six documents from four countries that fulfilled our inclusion criteria: two evidence-based guidelines [from the UK (22) and Australia (23)] and four national [UK (24), Republic of Ireland (25)] or regional [Canada/Ontario (26), Australia/Western Australia (27)] care models/pathways. **Table 2** gives an overview of the included documents.

**Table 2:** Overview of included documents.

Country (region, if applicable)	Title of document	Publisher	Year of publication	Type of document	Reference(s)
UK	Antenatal and postnatal mental health + Quality Standard	National Institute for Health and Care Excellence (NICE)	2014, updated 2020	Clinical guideline	(22, 28, 29)
UK	The Perinatal Mental Health Care Pathways + Implementation Guidance	National Health Service England (NHS), National Collaborating Centre for Mental Health (NCCMH)	2018	Guidance document	(13, 24)
Ireland	Specialist Perinatal Mental Health Services - Model of Care for Ireland	National Mental Health Division, Health Service Executive (HSE)	2017	NR	(25)
Canada (Ontario)	Care Pathway for the Management of Perinatal Mental Health + Guidance Document	Provincial Council for Maternal and Child Health (PCMCH)	2021	Guidance document	(26, 30)
Australia	Mental Health Care in the Perinatal Period: Australian Clinical Practice Guideline	Centre of Perinatal Excellence (COPE)	2017 (update currently in progress)	Clinical guideline	(23)
Australia (WA)	Perinatal and Infant Mental Health Model of Care – a framework	Western Australian Department of Health (WA DOH)	2016	NR	(27)

NR, not reported; UK, United Kingdom; WA, Western Australia.

Regarding the *development process*, all documents state that they have been developed by some form of working group including experts from various disciplines and professions. In five documents (22–25, 27), people with lived experience were also involved in the development of the care models. The two guidelines (22, 23) are described as being based on the best available evidence, prepared in the form of a systematic review. The other documents also state that the development of the care model was informed by evidence (24–27).

The *target users* of the documents include healthcare professionals caring for women and families in the perinatal period (22, 23, 26, 27) (e.g., mental health practitioners, midwives, general practitioners [GP], obstetricians, pediatricians), people responsible for service planning (22, 24, 27), as well as families and carers (22–24).

### Quality assessment of the included documents

The two guidelines (22, 23) scored 149 and 150 points respectively out of a possible 161 points, using the AGREE II tool, which corresponds to 93%. The adapted AGREE II tool, which we used for assessing the other documents, resulted in scores between 43 and 61 points out of a possible 77 points, which corresponds to 56-79%. The main limitations of the documents were missing information on evidence search methods and on the link between evidence and recommendations (24–27). The quality assessment tables can be found in the **Supplementary Material**.

### Characteristics of the described PIMH care models and pathways

#### Target populations

The target populations for the services described in the care models and guidelines are either all pregnant and postpartum women as well as women planning a pregnancy (22, 23, 26, 27), or those in the same stage of life with a past or current (suspected) mental health problem or at risk of developing a mental health problem (24, 25). Only one document also explicitly mentions fathers and fathers-to-be (27). Another document uses the term ‘pregnant and postpartum individuals’ to describe their target population (26). None of the documents explicitly addresses LGBTQI+ families or non-birthing parents.

#### Involved professionals

A range of involved professionals were mentioned in the included documents. These were categorized into three groups:

▪ Medical specializations:

• GPs
• Obstetricians
• Neonatologists
• Pediatricians
• Psychiatrists

▪ Midwifery and nursing specializations:

• Midwives
• Perinatal mental health midwives
• Maternal and child health nurses
• Mental health nurses
• Community psychiatric nurses
• Practice nurses
• Public health nurses
• Community nurses

▪ Allied professionals:

• Clinical psychologists
• Occupational therapists
• Social workers
• Peer support workers
• Health visitors

#### Education and training

Five documents (22–25, 27) emphasize the importance of appropriate training and supervision for health professionals working in perinatal mental healthcare. Documents state that training should cover, e.g., mental health problems, assessment methods and tools, and referral pathways (22, 23). The Australian COPE guidelines includes a consensus statement that ”all health professionals providing care in the perinatal period should receive training in woman-centered communication skills, psychosocial assessment and culturally safe care” (23, p.26). Training and supervision should not only be available to those healthcare professionals involved in the care of PMI, but for all those who care for pregnant and postpartum women (23, 25), for example regarding woman-centered communication skills, psychosocial assessment, and culturally safe care (23). Documents do not provide details of format of recommended training (e.g. online, in person) or length. Although some discuss training they have developed in the use of their guidelines, which can be accessed online.

#### Organization of services

All documents provide information (with different levels of detail) regarding the organization of the services. Generally, several different healthcare professionals and levels of care are involved in PIMH care. Universal services are those that all families have access to, including primary care, maternity services and health visits (24, 25, 27). Targeted services (at primary or secondary level) focus on women, children and families with additional needs or increased risk of poor health (27). Mental health services and specialist perinatal mental health services provide interventions to women and families with mental health problems. Documents recommend to organize those e.g., in clinical networks, linking community perinatal mental health teams and inpatient mother-baby-units (MBUs), as in the UK (22, 24). Another example is the ‘hub and spoke’ clinical network model from Ireland (25), with ‘hub’ hospitals in each hospital group (i.e. more centralized units with a higher level of expertise) and smaller units (‘spokes’) that are linked to one of the larger ‘hub’ hospitals for training, clinical advice and regular meetings. Each ‘hub’ should have a specialist perinatal mental health service with multidisciplinary staffing, and in each ‘spoke’ units, the liaison psychiatry team should be complemented by a mental health midwife. Clearly defined care pathways, as well as stepped-care approaches or frameworks, are considered as important and helpful for the organization and provision of PIMH care (22, 23, 26). Furthermore, the need for multidisciplinary networks is emphasized to have access to specialist expert advice, e.g., on medication during pregnancy and breastfeeding, to ensure transfer of information and continuity of care, and to help healthcare professionals navigate diverse treatment options (22, 23, 26).

#### ‘Components’ of care

**Figure 1:**
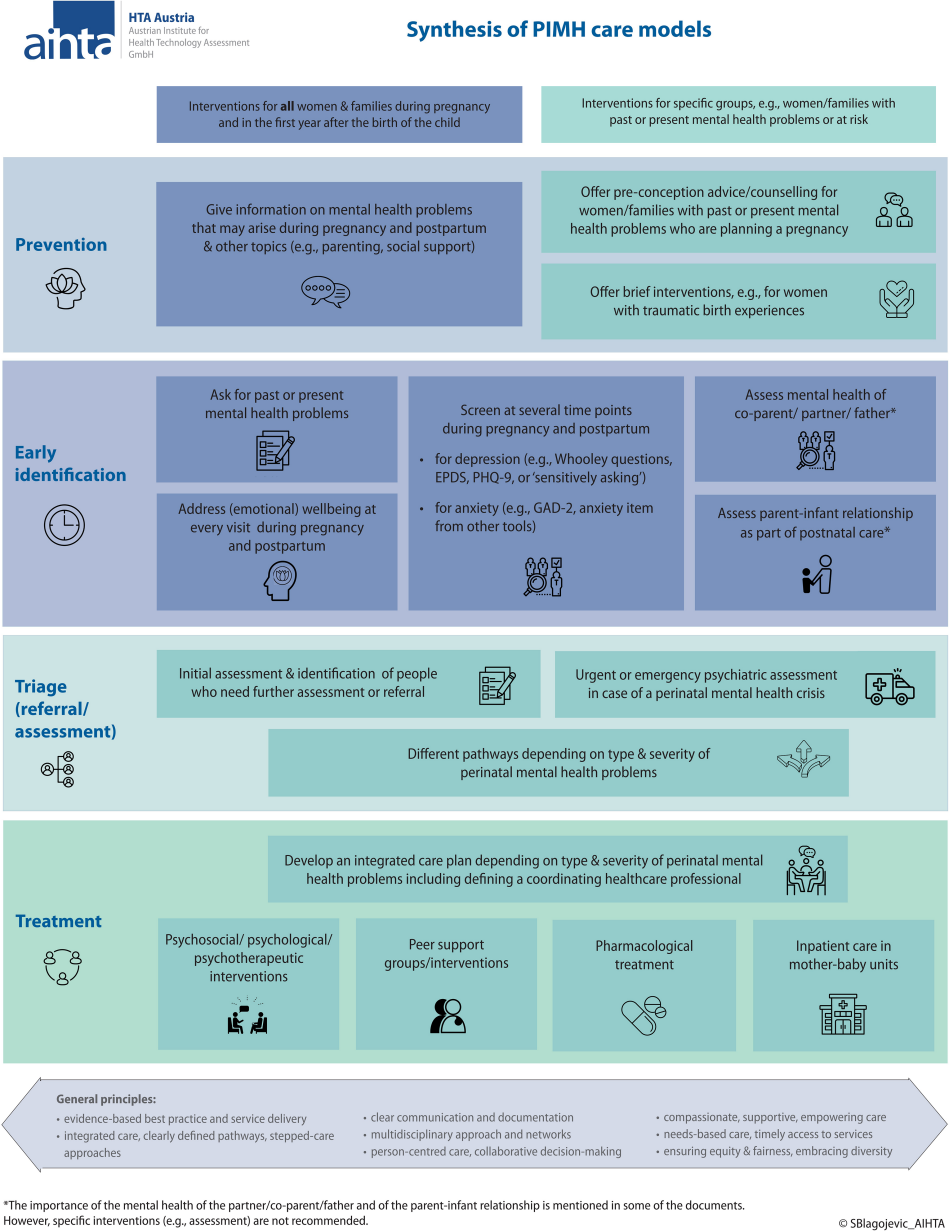
Synthesis of PIMH care models. EPDS, Edinburgh Postnatal Depression Scale; GAD-2, Generalized Anxiety Disorder-2; PHQ-9, Patient Health Questionnaire-9; PIMH, perinatal and infant mental health.

All included guidelines and care models provided information on the components of care, i.e., primary prevention, early identification, referral and assessment, and treatment. We also extracted information on specific services addressing parent-infant relationship and mental health of the partner/father/co-parent. The synthesis of the results is now summarized (also see **Figure 1**).

Regarding *primary prevention*, the documents recommended providing information on mental health in general and problems which may arise during pregnancy and after birth (e.g., emerging signs and symptoms of PMI) as well as on other relevant topics (such as social support, parenting issues or infant development) to all women, families, and expectant parents. Various dissemination methods can be used, e.g., print material, verbal information or home visits (22, 23, 27). More specifically, for women with new, pre-existing or past mental health problems or at risk for mental illness who are planning a pregnancy, pre-conception advice or planning is recommended in three documents (22, 24, 26). This includes giving information (e.g., on medication and breastfeeding) and optimizing management (e.g., improving health behaviors such as nutrition, physical activity or sleeping; reviewing psychological or pharmacological treatment). In the postpartum period, primary prevention may also include the offer of brief interventions, e.g., for women who experienced complications during pregnancy and/or birth or had traumatic births (25, 27). In Ireland, this can be one of the tasks of the so-called perinatal mental health midwives, who work with midwives and obstetricians at all levels of care (25).

*Early identification* of people with PMI is a crucial component of PIMH care. Screening of mothers for PMI is recommended by all included documents (except for the UK Pathways (24), which focus more on care pathways for already identified mental health problems). The Irish model of care (25) only mentions the mental health screening at the booking visit at the beginning of pregnancy and does not contain information on postpartum screening. The other documents recommend screening at the beginning of pregnancy (22, 23, 27), at all contacts during pregnancy and postnatal period (22, 26) or to repeat screening at least once later in pregnancy, at 6–12 weeks after birth and at least once later in the first year after the birth (23).

Several screening methods for depression are mentioned in the documents: the ‘Whooley questions’ (22, 25), the ‘Edinburgh Postnatal Depression Scale’ (EPDS) (22, 23, 27), the ‘Patient Health Questionnaire-9’ (PHQ-9) (22) as well as ‘sensitively asking’ without using a specific tool (26). The ‘Generalized Anxiety Disorder-2’ (GAD-2) or the anxiety items from other tools can be used to screen for anxiety disorders which is recommended by the two guidelines (22, 23). Guidelines themselves do not include details of validation of these screening tools, and many tools have been reported in the literature screening, with the EDPS being the most frequently validated tool (31). A recent review has been published regarding appropriateness of screening tools for fathers (32). Furthermore, asking for past or present mental health problems should be included in appointments in early pregnancy (22, 25). If alcohol misuse is suspected, the ‘Alcohol Use Disorders Identification Test’ (AUDIT) can be applied (22). Asking about the woman’s (emotional) wellbeing at every antenatal and postnatal visit is also recommended (23, 26).

Screening is recommended to be carried out during the ‘booking visit’ by the midwife or GP (22, 25), or wherever a woman seeks antenatal and postnatal care, e.g., in general practice (GP, practice nurse), in midwifery and maternal and child healthcare (midwife, maternal and child health nurse), or in obstetric practice (obstetrician, practice midwife) (23). Three documents do not specify which healthcare professional should conduct the screening (24, 26, 27).

*Triage* in mental health includes an initial assessment and the identification of people who need further assessment or referral. Different pathways were identified, depending on the type and severity of PMI (22–26). The Canadian (Ontario) guide has the following cut-off scores for assessment and triage (26, 30):

▪ Mild: PHQ-9 or GAD-7 score of 5-9, or EPDS score of 10 to 12.
▪ Moderate: PHQ-9 or GAD-7 score of 10–14 or EPDS score of 13 to 18.
▪ Severe: PHQ-9 or GAD-7 score greater than 15, EPDS score greater than 19 or question 10 has a score greater than zero.
▪ Urgent: active intent to harm self or others and/or suicidal ideation endorsed on any of the afore-mentioned questionnaires.

The Irish care model recommends that women with mild depression or anxiety symptoms should be referred to a mental health midwife, whereas women with moderate or severe PMI should be assessed by the specialist perinatal mental health services (25). In the case of a perinatal mental health crisis (e.g., risk of psychosis or suicide), an urgent or emergency psychiatric assessment is needed, especially if there is uncertainty about whether a woman could develop mania or psychosis, or if there are safety concerns about the woman or her family including infant (24, 26). Referral and assessment pathways also depend on the setting, e.g., GP or maternity setting and location, e.g., urban or rural. However, coordinated care and clear communication are recommended as essential in any case (23). In the UK, there are five pathways of care depending on the situation, including: (1) preconception advice, (2) specialist assessment, (3) emergency assessment, (4) psychological assessment, (5) urgent admission to an MBU (13, 24).

*Treatment* of PMI includes mainly psychosocial, psychological, psychotherapeutic interventions [e.g., structured psychoeducation, guided self-help, social support groups, directive counseling, cognitive behavioral therapy, interpersonal therapy; depending on the severity of the symptoms (22, 23, 26)], and pharmacological treatment (22, 23). Treatment options that are available when breastfeeding should also be discussed (22, 23). Both guidelines (22, 23) include recommendations on pharmacological treatment of certain perinatal mental health illnesses. However, as these recommendations are very specific, we refrained from extracting them into our tables, but we provide the reference and page number for further information (the full extraction tables are available from the authors). If inpatient care is required, women should have timely access to MBUs in the hospital. Staff with specialist expertise to manage complex or severe PMI are needed (24, 26). A stepped-care model of service delivery is recommended (22, 26, 27). Generally, treatment options should be discussed and planned together with the woman, her partner and family, leading to an individual care plan (26, 27).

Five documents mention services such as *peer support* groups or other interventions involving people with lived experience; however, detailed information are not provided (22, 23, 25–27). Women with PMI (especially with only mild or subclinical symptoms) should be informed of and could benefit from social support groups facilitated by peer volunteers who bring their lived-experience of PMI (22, 23, 26).

Information regarding services that address *infant (mental) health and/or parent-infant relationship* was found in four documents (22, 23, 25, 27). It is recommended to also assess the parent-infant relationship as part of postnatal care and to discuss any parental concerns. Documents describe that mental illness in one family member affects the wellbeing of the whole family, therefore they argue that it is crucial to provide integrated care that includes and considers the needs of all family members. Some documents also give examples for interventions aiming to improve the parent-infant interaction (25, 27).

In the included care models, all services are mainly focused on the (expectant) mothers. The mental health of the *co-parent/partner/father* is briefly addressed in some of the documents (22–24), but specific services are not mentioned.

Regarding *(cross-sectoral) coordination* of services, it is recommended to develop an integrated care plan including defining a coordinating healthcare professional (e.g., nurse, midwife) who ensures sharing of information with all services and people involved, continuity of care, and timely provision of the interventions (22, 25, 27).

## Discussion

This scoping review summarizes the common characteristics of six PIMH care models from four countries. The synthesis of the information on the various care components from the documents is visualized in **Figure 1**. The results highlight the need for integrated care models (including prevention, early identification and treatment), multi-professional networks, stepped-care approaches, clear referral pathways, and cross-sectoral coordination.

However, there remain a number of open questions which, in our view, have not been adequately addressed in the documents identified. These include the identification and care of fathers/co-parents/partners with PMI, the explicit inclusion and consideration of the (mental) well-being of the infant as well as other children in the family, and the specific role of people with lived experience.

PMI prevention and care should ideally consider the parents, the child, the parent-child relationship, and the family situation as a whole (including co-parents, other children, etc.). Parental mental health problems impact the child’s wellbeing and mental health, and the infant’s mental health affects parental well-being (11, 14, 33). Although some of the documents included emphasize the importance of the parent-child relationship, there is a lack of specific recommendations, e.g., on how to recognize and treat parent-child relationship disorders. A recently published systematic review identified models that integrate maternal mental health treatment into pediatric primary care or child mental health and concluded that better integration of care would likely improve child psychopathology, maternal mental health, and family function (34).

The identified care models focus mainly on the mothers: only one document explicitly mentions (expectant) fathers as a target group. The predominant focus on the mental health of the woman and the mother-child relationship neglects the importance of the mental health and well-being of the father/co-parent/partner. This focus also risks implicitly reproducing traditional gender roles and the view that pregnancy and childcare are primarily the responsibility of women. Therefore, more studies on fathers’ mental health, and tailored screening and intervention approaches are needed; and future research on PMI prevention and treatment should include the family system and move beyond the heteronormative construction of parenthood and the perinatal period (35, 36).

However, the revised COPE guideline, which was published after the literature search for this scoping review was completed, now includes recommendations addressing the mental health of non-birthing parents (e.g., fathers): it is recommended to offer mental health screening and psychosocial assessment as early as practical in pregnancy and 3–6 months after the birth with repeated screening when clinically indicated. Interestingly, the recommendations on the assessment of the parent-infant interaction still exclusively refer to the mothers. It has to be noted that all recommendations regarding mental health assessment for fathers and non-birthing parents are consensus-based recommendations, thus ‘formulated in the absence of quality evidence’ (37).

Several documents emphasize the need for education and training for healthcare professionals involved in the care of pregnant and postpartum women and families. Supervision is also an important topic because of the emotional challenges of working with women and families with PMI. Many professionals are highly motivated but underestimate the emotional challenges. Additionally, they may have experiences and competence in working with adults, but not with babies and infants, and vice versa (27). Reflective supervision is considered to help prevent burn-out and promote work satisfaction. However, more research is needed to strengthen the evidence base on proposed outcomes of reflective supervision in the field of infant mental health (38). In a recent European survey, a considerable gap was identified between the expected skills and the available training for psychiatrists regarding perinatal mental health. Urgent action is needed, given the high prevalence and impact of PMI, as well as the expressed wishes for specialist training by the professionals themselves (39). Knowledge and health literacy regarding perinatal mental health are also low among perinatal women and the general public, as was shown in a systematic review. The authors concluded that campaigns and interventions to improve perinatal mental health literacy are needed (40).

Another scoping review mapped the state of perinatal mental health care in the WHO Europe Region. The following countries were identified as leading countries which can serve as models for other countries: Belgium, Finland, Ireland, Netherlands, Sweden, UK, and Malta. However, in most countries in the WHO Europe Region, PMH is still at an early stage of development (41). The WHO provides guidance for countries to establish and improve their PMH services (42). Although this study is situated in a regional context, the challenges and strategies identified, particularly regarding fragmented services and inter-sector collaboration, are shared across many health systems globally. As such, the findings offer transferable insights for international efforts to strengthen perinatal and infant mental health care.

We are aware of the following limitations of our review: First, we only included documents written in English or German language; however, with our search methods, we did not identify any specific documents in other languages with comparable scope and content as the documents that we included. Some countries may have comprehensive care models in place without having a written guideline or best-practice model; these implemented practices that do not have corresponding written documents would have been missed. Further, we refrained from conducting a systematic literature search, as those care models and pathways are usually not published in scientific journals (‘grey literature’). Instead, we comprehensively searched topic-specific websites and also asked several experts for relevant documents.

## Conclusions

Based on our results, the ‘ideal’ care model for PIMH should be evidence-based, needs-based, person-centered, and equitable, and provide compassionate, supportive, empowering care, based on collaborative decision-making. It should include integrated pathways and multi-professional, coordinated networks, and include interventions of primary prevention, counseling, and effective early identification and screening. The ‘ideal’ care model should have clearly defined referral pathways and stepped-care approaches and provide appropriate evidence-based treatment with timely access. It considers not only the mental health and wellbeing of the mother, but also of the child(ren) and the father/partner/co-parent, as well as the parent-infant relationship. People with lived experiences are involved when designing and implementing PIMH, but also when supporting people with PMI e.g., by providing peer-support groups. Evaluation and/or monitoring of newly implemented interventions should be planned from the beginning.

The results from this scoping review can be used for further discussion and as decision support for designing, developing and implementing PIMH care. Within the research project ‘Co-designing perinatal mental health support in Tyrol’, the results will be used to inform the participatory development of a support intervention which will subsequently be implemented and evaluated. This study underscores the need for integrated, participatory models of care that are responsive to local contexts yet guided by shared principles, such as sustainability, early intervention, and supporting development of services with lived experience. Future work should build on these foundations to co-design sustainable systems of support for families.

## Author’s note

The content of this manuscript has been presented in part as a report at the Austrian Institute for Health Technology Assessment (Reinsperger I, Paul J. Perinatal and infant mental health care models and pathways. A scoping review. AIHTA Project Report No.: 148; 2022. Vienna: HTA Austria – Austrian Institute for Health Technology Assessment GmbH. https://eprints.aihta.at/1420/).
