# Supplementary material for: International best-practice models for perinatal and infant mental health care – a scoping review
Source: Front Psychiatry. 2025 Jun 17;16:1536145. doi: 10.3389/fpsyt.2025.1536145 (PMC12209273; doi:10.3389/fpsyt.2025.1536145)
Supplement: Supplementary file 1 [file Table1.docx]

**Supplementary Material**

LITERATURE SEARCH

In addition to the databases, we searched the websites of the following guideline developing institutions:

- National Institute for Health and Care Excellence (NICE),
- Scottish Intercollegiate Guidelines Network (SIGN),
- World Health Organization (WHO),
- Association of the Scientific Medical Societies (*Arbeitsgemeinschaft der Wissenschaftlichen Medizinischen Fachgesellschaften*, AWMF),
- U.S. Preventive Services Task Force (USPSTF),
- Canadian Task Force on Preventive Health Care (CTFPHC),
- Royal Australian and New Zealand College of Psychiatrists (RANZCP),
- Royal Australian and New Zealand College of Obstetricians and Gynaecologists (RANZCOG).

For identifying documents that describe care models or pathways, we additionally searched on the websites of the following institutions:

- Maternal Mental Health Alliance (MMHA),
- Marcé Society for Perinatal Health,
- Global Alliance for Maternal Mental Health,
- Perinatal Anxiety & Depression Australia (PANDA).

After a general search for models and pathways (without country restrictions), we carried out a more specific hand search for relevant documents from certain countries. We searched the websites of the respective national ministries of health and public health institutions. Within Europe, we focused the specific search for relevant documents on countries that were ranked in the Top 30 of the ‘Human Development Index’ (HDI)^[[1]](#footnote-1)^ and had more than five million inhabitants^[[2]](#footnote-2)^.

QUALITY ASSESSMENT

**Selected items** from the AGREE II Tool to assess the quality of (guidance) documents describing PIMH care models:

- Item 1: The overall objective(s) of the [document] is (are) specifically described.
- Item 3: The population (patients, public, etc.) to whom the [document] is meant to apply is specifically described.
- Item 4: The [document] development group includes individuals from all the relevant professional groups.
- Item 5: The views and preferences of the target population (patients, public, etc.) have been sought.
- Item 6: The target users of the [document] are clearly defined.
- Item 7: Systematic methods were used to search for evidence.
- Item 12: There is an explicit link between the recommendations and the supporting evidence.
- Item 17: Key recommendations are easily identifiable.
- Item 18: The [document] describes facilitators and barriers to its application.
- Item 20: The potential resource implications of applying the recommendations have been considered.
- Item 21: The [document] presents monitoring and/or auditing criteria.

**Quality assessment results**

see Table 1.

Table 1: Quality assessment of the included guidelines and other documents

| Quality Assessment Check | COPE, 2017 | NICE, 2014 | UK,  2018 | Ireland,  2017 | Canada/ Ontario, 2021 | Australia/ WA, 2016 |
| --- | --- | --- | --- | --- | --- | --- |
| Domain 1: Scope and Purpose |  |  |  |  |  |  |
| 1. The overall objective(s) of the guideline [document] is (are) specifically described. | 7 | 7 | 7 | 6 | 7 | 6 |
| 2. The health question(s) covered by the guideline is (are) specifically described. | 7 | 7 | n.a. | n.a. | n.a. | n.a. |
| 3. The population (patients, public, etc.) to whom the guideline [document] is meant to apply is specifically described. | 7 | 7 | 7 | 5 | 4 | 6 |
| Domain 2: Stakeholder Involvement |  |  |  |  |  |  |
| 4. The guideline [document] development group includes individuals from all the relevant professional groups. | 7 | 7 | 7 | 7 | 2 | 7 |
| 5. The views and preferences of the target population (patients, public, etc.) have been sought. | 5 | 7 | 7 | 7 | 1 | 3 |
| 6. The target users of the guideline [document] are clearly defined. | 7 | 7 | 7 | 5 | 4 | 5 |
| Domain 3: Rigour of Development |  |  |  |  |  |  |
| 7. Systematic methods were used to search for evidence. | 7 | 7 | 5 | 1 | 7 | 1 |
| 8. The criteria for selecting the evidence are clearly described. | 7 | 7 | n.a. | n.a. | n.a. | n.a. |
| 9. The strengths and limitations of the body of evidence are clearly described. | 7 | 7 | n.a. | n.a. | n.a. | n.a. |
| 10. The methods for formulating the recommendations are clearly described. | 7 | 7 | n.a. | n.a. | n.a. | n.a. |
| 11. The health benefits, side effects, and risks have been considered in formulating the recommendations. | 7 | 7 | n.a. | n.a. | n.a. | n.a. |
| 12. There is an explicit link between the recommendations and the supporting evidence. | 7 | 7 | 1 | 1 | 5 | 3 |
| 13. The guideline has been externally reviewed by experts prior to its publication. | 5 | 7 | n.a. | n.a. | n.a. | n.a. |
| 14. A procedure for updating the guideline is provided. | 6 | 7 | n.a. | n.a. | n.a. | n.a. |
| Domain 4: Clarity of Presentation |  |  |  |  |  |  |
| 15. The recommendations are specific and unambiguous. | 7 | 6 | n.a. | n.a. | n.a. | n.a. |
| 16. The different options for management of the condition or health issue are clearly presented. | 7 | 7 | n.a. | n.a. | n.a. | n.a. |
| 17. Key recommendations are easily identifiable. | 7 | 6 | 6 | 5 | 5 | 7 |
| Domain 5: Applicability |  |  |  |  |  |  |
| 18. The guideline [document] describes facilitators and barriers to its application. | 7 | 1 | 1 | 4 | 3 | 6 |
| 19. The guideline provides advice and/or tools on how the recommendations can be put into practice. | 7 | 7 | n.a. | n.a. | n.a. | n.a. |
| 20. The potential resource implications of applying the recommendations have been considered. | 5 | 3 | 6 | 6 | 4 | 2 |
| 21. The guideline [document] presents monitoring and/or auditing criteria. | 3 | 7 | 7 | 5 | 1 | 4 |
| Domain 6: Editorial Independence |  |  |  |  |  |  |
| 22. The views of the funding body have not influenced the content of the guideline. | 7 | 7 | n.a. | n.a. | n.a. | n.a. |
| 23. Competing interests of guideline development group members have been recorded and addressed. | 7 | 7 | n.a. | n.a. | n.a. | n.a. |
| Overall quality of this guideline/document  (1 – lowest possible quality, 7 – highest possible quality) | 150 of 161 (93%) | 149 of 161 (93%) | 61 of 77 (79%) | 52 of 77 (68%) | 43 of 77  (56%) | 50 of 77 (65%) |

Abbreviations: COPE – Centre for Perinatal Excellence, NICE – National Institute for Health and Care Excellence, UK – United Kingdom, WA – Western Australia

1. <https://en.wikipedia.org/wiki/Human_Development_Index>, accessed 21/10/2022 [↑](#footnote-ref-1)
2. Countries that fulfil those criteria are: Norway, Sweden, Denmark, Finland, UK, Ireland, Switzerland, Germany, Netherlands, Belgium, France, Spain, Italy, Czech Republic (and Austria). [↑](#footnote-ref-2)
